# Visualization of Double-Stranded RNA Colocalizing With Pattern Recognition Receptors in Arenavirus Infected Cells

**DOI:** 10.3389/fcimb.2018.00251

**Published:** 2018-07-24

**Authors:** Elizabeth J. Mateer, Slobodan Paessler, Cheng Huang

**Affiliations:** Department of Pathology and Institute for Human Infections and Immunity, University of Texas Medical Branch, Galveston, TX, United States

**Keywords:** dsRNA, negative-sense RNA virus, arenaviruses, Junín virus, pattern recognition receptor, RIG-I, MDA-5, PKR

## Abstract

An important step in the initiation of the innate immune response to virus infection is the recognition of non-self, viral RNA, including double-stranded RNA (dsRNA), by cytoplasmic pattern recognition receptors (PRRs). For many positive-sense RNA viruses and DNA viruses, the production of viral dsRNA, and the interaction of viral dsRNA and PRRs are well characterized. However, for negative-sense RNA viruses, viral dsRNA was thought to be produced at low to undetectable levels and PRR recognition of viral dsRNA is still largely unclear. In the case of arenaviruses, the nucleocaspid protein (NP) has been identified to contain an exoribonuclease activity that preferentially degrades dsRNA in biochemical studies. Nevertheless, pathogenic New World (NW) arenavirus infections readily induce an interferon (IFN) response in a RIG-I dependent manner, and also activate the dsRNA-dependent Protein Kinase R (PKR). To better understand the innate immune response to pathogenic arenavirus infection, we used a newly identified dsRNA-specific antibody that efficiently detects viral dsRNA in negative-sense RNA virus infected cells. dsRNA was detected in NW arenavirus infected cells colocalizing with virus NP in immunofluorescence assay. Importantly, the dsRNA signals also colocalized with cytoplasmic PRRs, namely, PKR, RIG-I and MDA-5, as well as with the phosphorylated, activated form of PKR in infected cells. Our data clearly demonstrate the PRR recognition of dsRNA and their activation in NW arenavirus infected cells. These findings provide new insights into the interaction between NW arenaviruses and the host innate immune response.

## Introduction

An essential aspect of the innate immune response is the activation of pattern recognition receptors (PRRs). During viral infections, PRRs can recognize pathogen-associated molecular patterns (PAMPs), which leads to the induction of an interferon (IFN) response or inhibition of virus translation (Jensen and Thomsen, 2012). Double-stranded (ds) RNA is one such PAMP produced during virus replication that can be recognized by the cytoplasmic PRRs dsRNA-dependent protein kinase R (PKR) or the retinoic acid-inducible gene I (RIG-I)-like receptors (RLRs) (Gantier and Williams, 2008; Lemaire et al., 2008; Jensen and Thomsen, 2012). Two dsRNA binding domains on PKR mediate PKR recognition of dsRNA (usually longer than 33 bp). Subsequently, PKR undergoes dimerization and autophosphorylation, and becomes enzymatically activated. The phosphorylated, activated form of PKR, p-PKR, can then phosphorylate the translation initiation factor eIF2α leading to the inhibition of host and/or viral translation (Lemaire et al., 2008). The RLRs, RIG-I and melanoma differentiation-associated antigen 5 (MDA-5), consist of a C-terminal ssRNA/dsRNA or dsRNA binding domain, respectively (Jensen and Thomsen, 2012). RLR binding of non-self, viral RNA species and ATP triggers signal transduction that ultimately leads to the translocation of IRF3 and IRF7 to the nucleus and the transcription of IFN genes and the IFN-stimulated genes. The substrate preference for RLR recognition is still not fully understood. In general, RIG-I has a substrate specificity for 5′ triphosphate ssRNA or dsRNA, whereas MDA-5 recognizes longer dsRNA species (Jensen and Thomsen, 2012). Many viruses have evolved different strategies to evade PRR recognition by either hiding dsRNA from recognition, masking dsRNA through termini processing, or directly interacting with the RLRs or downstream components of the RLR activation pathway in order to suppress the innate immune responses (Zinzula and Tramontano, 2013).

Arenaviruses are single-stranded, negative-sense RNA viruses with a bisegmented genome encoding four viral proteins by utilizing an ambisense coding strategy (Buchmeier et al., 2007). The 5′- and 3′-ends of arenavirus genomic RNA is critical for viral RNA replication and transcription, which forms a 5′-triphosphate (5′-ppp) containing a pan-handle structure (Buchmeier et al., 2007). A highly structured intergenic region separates two open reading frames on the viral genomic and antigenomic RNA and serves as transcription termination site. The L segment genomic RNA encodes the RNA-dependent RNA polymerase and the Z protein, similar to the matrix protein of other RNA viruses that drives virus particle formation and budding. The S segment encodes the nucleocaspid protein (NP) and the glycoprotein (Buchmeier et al., 2007).

The mammalian arenaviruses are classified into Old World (OW) and New World (NW) arenaviruses. The OW arenavirus Lassa virus (LASV) and the NW arenaviruses Junín virus (JUNV) and Machupo virus (MACV) are viral pathogens that can cause severe hemorrhagic fever disease in humans (McLay et al., 2014). JUNV is the causative agent of Argentine hemorrhagic fever (AHF) with a case fatality rate of 15–30% (Enria et al., 2008). The host innate immune response to arenavirus has been studied extensively in the past. The highly conserved DEDDh motif required for exonuclease activity is conserved in the NPs of all arenaviruses regardless of their pathogenicity and geographic origin (Qi et al., 2010; Hastie et al., 2011; Zhang et al., 2013; West et al., 2014; Huang Q. et al., 2015), suggesting its importance in the arenavirus life cycle. It has been proposed that the exoribonuclease activity could enable arenavirus to evade the innate immune response (Qi et al., 2010; Hastie et al., 2011; Huang Q. et al., 2015). In biochemical studies, the NPs of LASV, Lymphocytic choriomeningitis virus (LCMV), Tacaribe virus (TCRV), and Pichinde virus (PICV), but not JUNV, have been found to have a 3′-5′ exoribonuclease activity that preferentially digest dsRNA (Qi et al., 2010; Hastie et al., 2011; Zhang et al., 2013; West et al., 2014; Huang Q. et al., 2015). Mutations in the LASV NP DEDDh domain result in the activation of a type I IFN response in LASV infected cells (Reynard et al., 2014). Similarly, PICV with mutations in the NP DEDDh motif have been found to elicit a robust type I IFN response and is attenuated *in vivo* (Huang Q. et al., 2015). Together this suggest that the DEDDh motif is critical to prevent PRR recognition. Additionally, arenaviruses are able to mask dsRNA through termini processing. The prime and align strategy utilized for arenavirus RNA replication results in the addition of an unpaired G overhang at the 5′-ppp pan-handle structure (Marq et al., 2010, 2011). Model dsRNA molecules mimicking the unpaired 5′-ppp pan-handle structure of arenavirus are poor substrates for RIG-I recognition in biochemistry studies (Marq et al., 2010, 2011). Along with masking dsRNA from recognition by PRRs, the arenavirus NPs and the Z proteins of pathogenic arenaviruses have been shown to antagonize the IFN response by inhibiting RLRs and components of the RLR pathway in plasmid expression system or the surrogate, non-pathogenic arenavirus expression systems (Martínez-Sobrido et al., 2006, 2007; Fan et al., 2010; Zhou et al., 2010; Pythoud et al., 2012; Rodrigo et al., 2012; Koma et al., 2013; Xing et al., 2015).

On the other hand, severe and fatal AHF cases are associated with very high levels of serum IFN-α (2,000–64,000 IU/mL), which correlates with disease severity (Levis et al., 1984, 1985). NW arenavirus MACV also stimulates a potent IFN response in a non-human primate model (Stephen et al., 1977). We have previously shown that JUNV infection induced a RIG-I dependent type I IFN response in human cells, and that JUNVs are relatively insensitive to IFN treatment in human cells (Huang et al., 2012, 2014). Additionally, PKR, a known sensor of dsRNA, is activated during pathogenic JUNV and MACV infections without affecting virus replication (Huang et al., 2017). Another group, also reported that the vaccine strain JUNV infection leads to PKR phosphorylation, although the PKR mediated-eIF-2α phosphorylation was not detected (King et al., 2017a). Accordingly, we hypothesize that dsRNA are sensed by, and activate cytoplasmic PRR, during NW arenavirus infection.

The interaction between viral dsRNA and the PRRs can be studied by direct visualization of dsRNA and the PRR in a single cell with immunofluorescence staining. The widely used dsRNA-specific J2 or K1 monoclonal antibodies (MAb) have been successful for positive-sense RNA virus and DNA virus. However, a limitation could exist as the level of dsRNA produced during negative-sense RNA virus infection is generally below the detection limit using the same approach (Weber et al., 2006). Other options also include using specific RNA probes to detect viral RNAs (e.g., RNA FISH), but the target sequence must be known and might not be compatible with co-staining of proteins of interest. Son et al. found that the 9D5 MAb, which was developed initially for diagnosis of pan-enterovirus, had a high affinity for dsRNA and could detect viral dsRNA in negative-sense RNA virus infected cells, including dsRNA in LCMV-infected cells (Son et al., 2015). To further understand the mechanism of NW JUNV induced IFN response, we aimed to identify the potential interaction between NW arenavirus dsRNA and the cytoplasmic PRRs. By using the MAb 9D5 to detect dsRNA, we provide evidence that dsRNA is produced during NW arenavirus infection, and interacts with cytoplasmic PRRs, such as PKR, RIG-I and MDA-5.

## Materials and methods

### Cells and viruses

Human lung epithelial cells A549 (ATCC) were maintained in Dulbecco's modified Eagle's medium (Corning) supplemented with 10% heat inactivated fetal calf serum (Atlanta Bio) and 100 U/mL of streptomycin and penicillin (Hyclone). The recombinant Candid#1 (rCandid) vaccine strain of JUNV was generated as previously described (Emonet et al., 2011).

### Reagents

The antibody used to detect dsRNA was a murine MAb pan-Enterovirus clone 9D5 purchased from Millipore Sigma (cat#3361, ready for use, further diluted by dilution 1:2 in our lab). The rabbit MAbs against PKR (1:200, ab32506), p-PKR (1:100, ab81303) and MDA-5 (1:250, ab126630) were purchased from Abcam. The secondary antibodies, Goat-anti-mouse Alexa Fluor 488 (1:2,000) and Donkey-anti-rabbit Alexa Fluor 594 (1:2,000) were purchased from Invitrogen. The RIG-I antibody conjugated Alexa-594 was purchased from Santa Cruz (1:1,000, sc-376845). A murine monoclonal anti-JUNV NP antibody was obtained from BEI (NA05-AG12) and conjugated to Alexa 647 dyes (1:1,000, Invitrogen). RNase III was purchased from Life technologies (AM2290) and RNase I from Promega (M4261).

### Immunofluorescence staining

A549 cells were grown on PDL coated coverslips (Neuvitro) and infected with rCandid strain of JUNV at an MOI of 1. Forty-eight hours post infection (hpi), cells were fixed with 100% ice-cold methanol for 15 min at −20°C and then washed with PBS (Corning) at 4°C three times. Cells were permeabilized with 0.2% Triton X-100 (Sigma) for 5 min. For RNase treatment, 10 units of RNase I or III were incubated on coverslips for 20 min at room temperature. After RNase treatment or permeabilization, coverslips were washed 4 times in PBS. Primary antibodies were diluted in 3% BSA (Sigma) and incubated at 4°C overnight. Cells were then washed 5 times in PBS. Secondary antibodies were diluted in 3% BSA and incubated at room temperature for 1 h. Cells were washed 5 times in PBS. For triple immunofluorescence staining, conjugated antibodies were diluted in 3% BSA and incubated at RT for 2 h or 4°C overnight. Cells were washed five times in PBS. Cells were counterstained with 1 μg/mL DAPI in PBS. Cells were washed three times with PBS containing 0.05% Triton X-100, two times with PBS, and with ddH20 for one minute. Coverslips were inverted onto ProLong Gold antifade reagent (Invitrogen) on glass microscope slides (Fisher Scientific) then left to cure overnight. Coverslips were imaged on a Laser Confocal Scanning Olympus FV1000D Upright Microscope BX61 using 60x/1.42 numerical aperture oil immersion lens. Laser diode, visible argon ion, and helium neon lasers were used and emissions read using 385–405 nm (DAPI), 470–530 nm (Alexa 488), 560–615 nm (Alexa 594), and 615–690 nm (Alexa 647-conjugated). Unless indicated, laser emissions were the same for all samples. In **Figure 4B**, exposure was enhanced to acquire the RIG-I distribution pattern in mock-infected cells. All image analysis and processing was performed using the FIJI software and only the same linear adjustments for brightness and contrast were made when appropriate across all samples in same experiments.

## Results

### Identification of dsRNA in JUNV-infected human cells

**Figure 1:**
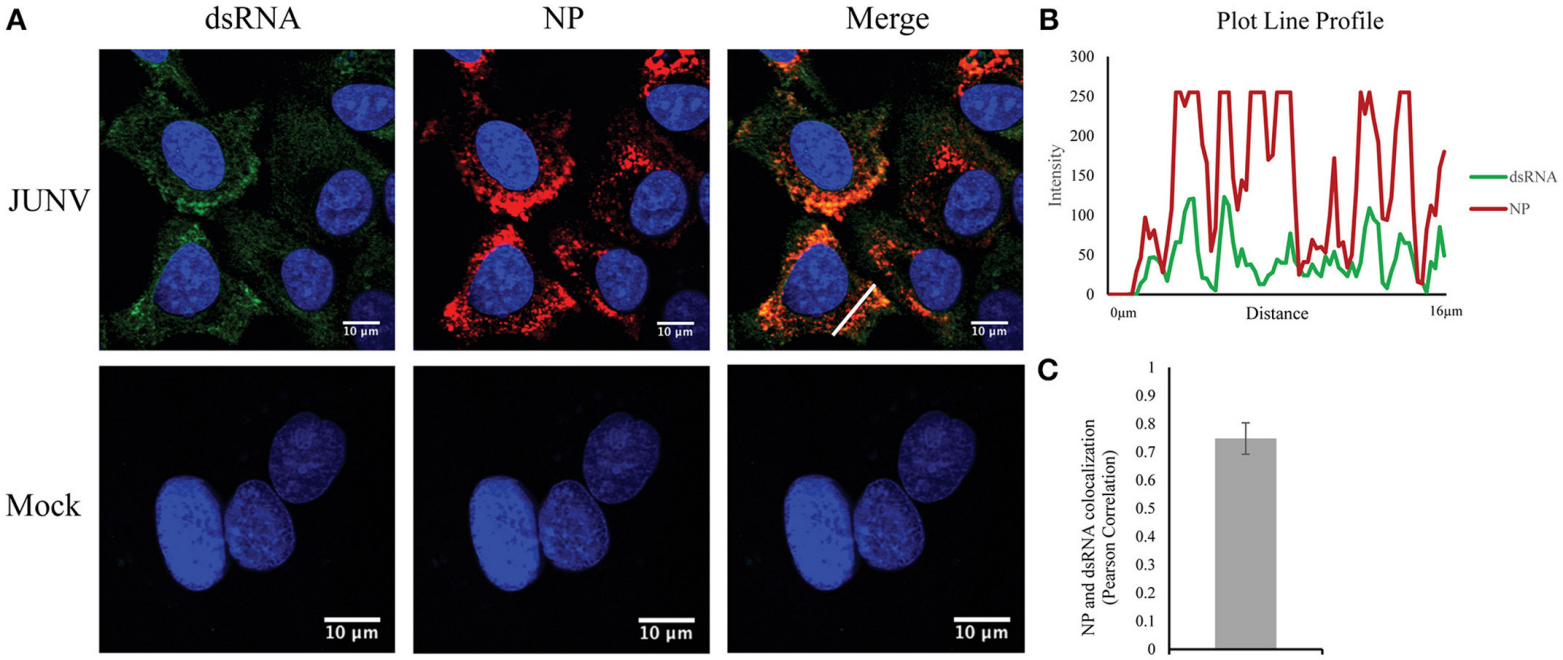
Detection of dsRNA in JUNV-infected A549 cells**. (A)** A549 cells were infected with the vaccine rCandid strain of JUNV at a MOI of 1.0. At 48 hpi, infected (JUNV) and uninfected (Mock) cell monolayers were fixed, permeabilized, and stained with the anti-dsRNA antibody (9D5, green), anti-JUNV NP (AG12, red) and DAPI (blue) as described in the Materials and Methods section. Cells were then observed under a Laser Confocal Scanning Olympus FV1000D Upright Microscope BX61 using 60x/1.42 numerical aperture oil immersion lens. All image analysis and processing was done using the FIJI software. Laser emissions were the same for all samples and only the same linear adjustments for brightness and contrast were made when appropriate across all samples. Data shown are representative images from three separate experiments. Fluorescence plot profile of NP and dsRNA signal intensities around the white line is shown in **(B)**. **(C)** Quantitative analysis of the colocalization between dsRNA and NP signals using the Pearson's correlation coefficient in 100 infected cells from three separate experiments. The average and Std are given.

Our previous studies have found that NW arenavirus infections activate both IFN response in a RIG-I dependent manner and the PKR response. Upon virus replication, cytoplasmic PRRs could recognize non-self, viral dsRNA and initiate the innate immune response (Gantier and Williams, 2008). The MAb 9D5 was originally used for diagnosis of pan-enterovirus, but turned out to be a dsRNA-specific antibody with an affinity for dsRNA higher than the widely used MAb J2 (Son et al., 2015). We, therefore, utilized the MAb 9D5 to determine if dsRNA was detectable during NW arenavirus JUNV infection. Our previous work revealed that both the pathogenic Romero strain and the vaccine Candid#1 strain of JUNV induce a type I IFN response *in vitro* (Huang et al., 2012). Therefore, the Candid#1 vaccine strain was utilized in this study to establish the method in BSL2 labs. Human lung epithelial A549 cells were infected with the rCandid vaccine strain of JUNV and immuno-stained for the presence of dsRNA and the viral NP. In mock infected cells, the dsRNA signal was very low or undetectable (Figure 1A). In comparison, in JUNV-infected cells, the dsRNA signals were readily detected and exhibited a diffused distribution in the cytoplasm with areas of concentrated dsRNA that colocalized with the punctuated staining of the NP (Figures 1A,B). The punctuated, perinuclear staining for the JUNV NP in infected cells is consistent with published work by other groups (Baird et al., 2012; King et al., 2017a). To quantify the colocalization of dsRNA and the NP signals, we randomly choose 100 infected cells in different fields from three independent experiments and measured the Pearson's correlation coefficients. The average Pearson's correlation coefficients was 0.75 (Figure 1C), suggesting a strong colocalization between dsRNA and NP signals (Zinchuk and Grossenbacher-Zinchuk, 2014). Overall, our data indicate the formation of dsRNA and its colocalization with the viral NP during JUNV infection.

### Specificity of the dsRNA antibody in JUNV-infected cells

**Figure 2:**
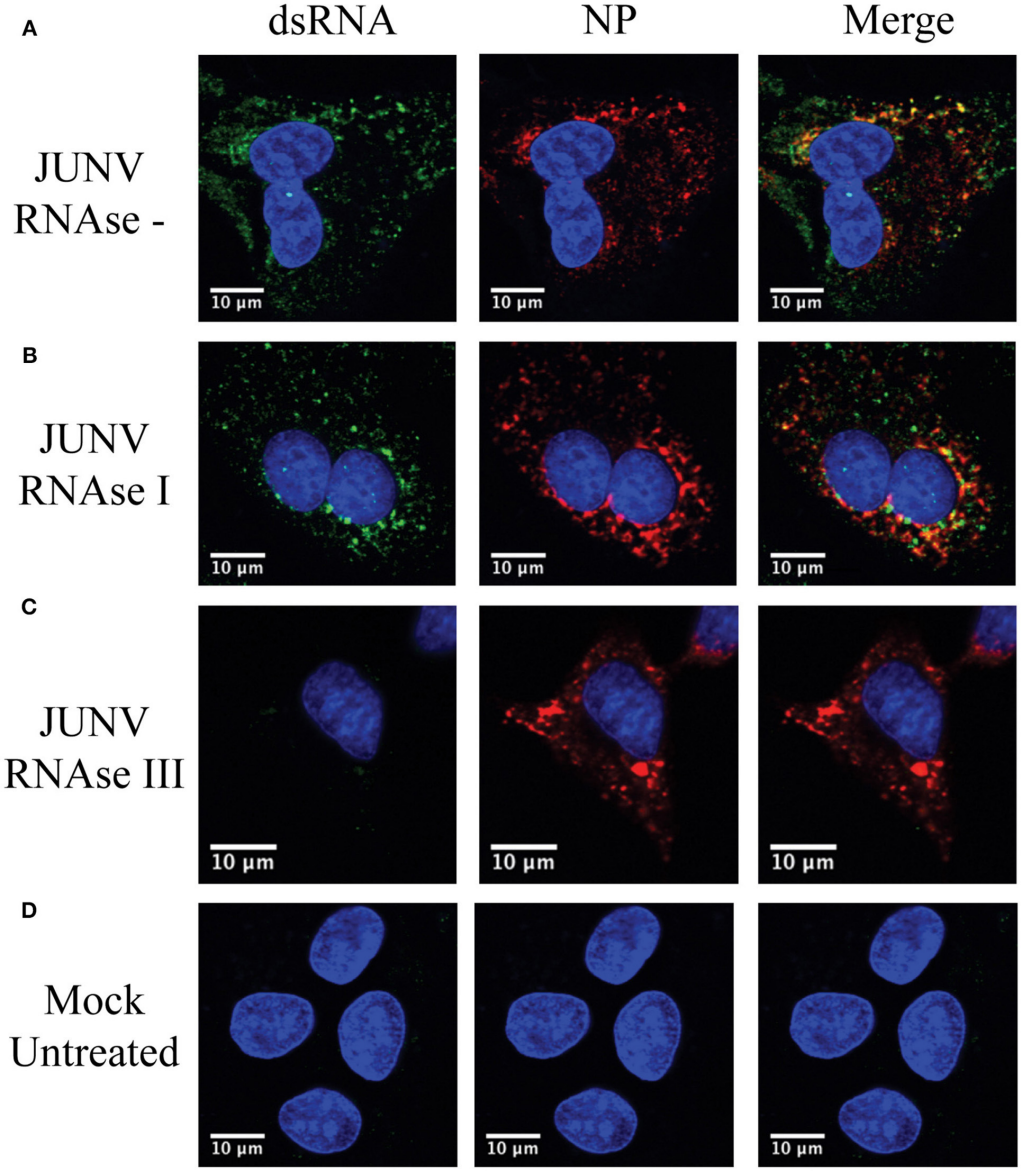
9D5 antibody specific for dsRNA. A549 cells were infected with JUNV at an MOI of 1.0, At 48 hpi, infected and uninfected cell monolayers were untreated **(A,D)** or treated with RNase I **(B)** and RNAse III **(C)**. **(A–D)** Cells were then stained with the anti-dsRNA antibody (9D5, green), anti-JUNV NP (AG12, red) and DAPI (blue) as described in the Materials and Methods section. Cells were then observed under a Laser Confocal Scanning Olympus FV1000D Upright Microscope BX61 using 60x/1.42 numerical aperture oil immersion lens. All image analysis and processing was done using the FIJI software. Laser emissions were the same for all samples and only the same linear adjustments for brightness and contrast were made when appropriate across all samples. Images shown are representative from three separate experiments.

To confirm that the MAb 9d5 was recognizing dsRNA, JUNV-infected A549 cells were treated with RNase III, which degrades dsRNA, or RNase I, which preferentially digests ssRNA. The RNase I treated samples retained similar levels of dsRNA staining compared to the untreated, infected samples (Figure 2). In contrast, the immunofluorescence staining for dsRNA in the RNase III treated samples was below the limit of detection (Figure 2), while the viral NP was still detected, as in infected cells without RNase treatment. This result confirmed that the MAb 9D5 specifically detected dsRNA in arenavirus infected cells.

### Activated PKR colocalizes with dsRNA in JUNV-infected cells

**Figure 3:**
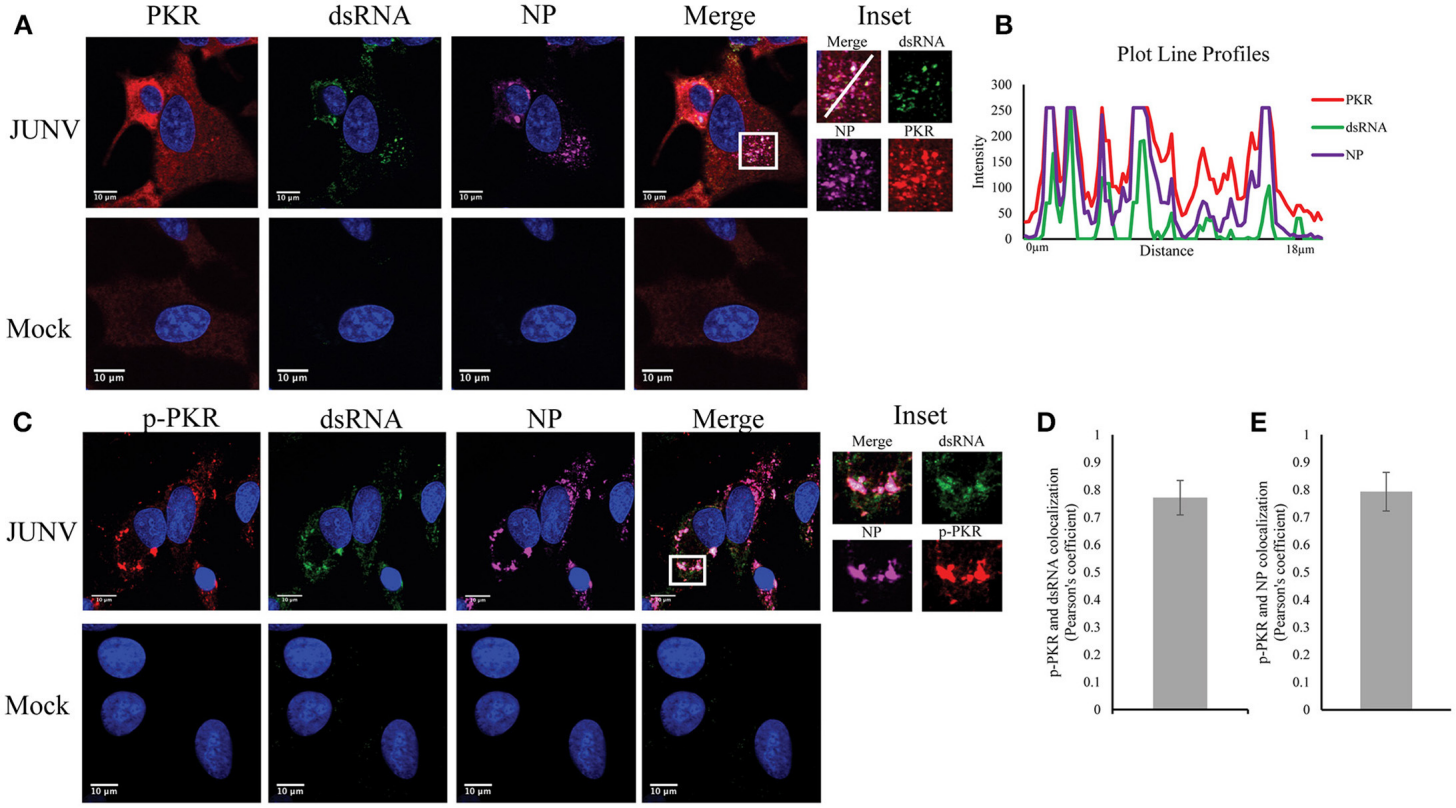
PKR recognizes JUNV dsRNA resulting in activation**. (A,C)** A549 cells were infected with rCandid at a MOI of 1.0. At 48 hpi, infected and uninfected cell monolayers were fixed, permeabilized, and stained with the anti-dsRNA antibody (9D5, green), anti-JUNV NP (AG12, magenta), anti-PKR (Y117, red) **(A)** or anti-p-PKR (Thr-451, red) **(C)** and DAPI (blue). Cells were then observed under a Laser Confocal Scanning Olympus FV1000D Upright Microscope BX61 using 60x/1.42 numerical aperture oil immersion lens. All image analysis and processing was done using the FIJI software. Laser emissions were the same for all samples and only the same linear adjustments for brightness and contrast were made when appropriate across all samples. **(B)** Fluorescence plot profile of PKR, NP and dsRNA signal intensities around the white line is shown in the inlet. **(D,E)** Quantification of the colocalization between dsRNA **(D)** or NP **(E)** and p-PKR using the Pearson's correlation coefficient. 100 infected cells from three separate experiments were randomly selected and analyzed. Data shown is the average and Std.

Upon dsRNA recognition, PKR undergoes autophosphorylation and becomes activated (Lemaire et al., 2008). We have previously shown that NW arenavirus JUNV and MACV infections activated PKR accompanied with suppression of host translation (Huang et al., 2017). Another group has also found that infection with the vaccine strain of JUNV leads to PKR phosphorylation (King et al., 2017a). Therefore, we sought to determine if dsRNA was recognized by, and consequently activated PKR during JUNV infection. A549 cells were infected with JUNV and stained for PKR or the activated form of PKR (p-PKR), dsRNA, and NP (Figure 3A). In mock infected cells, PKR was diffusedly distributed throughout the cytoplasm (Figure 3A), while in JUNV-infected cells, PKR formed areas of punctuated structures (Figure 3A inset), which colocalized with dsRNA and NP signals (Figure 3B). Furthermore, the phosphorylated, enzymatically active form of PKR could be detected in JUNV infected cells (Figure 3C), where it clearly colocalized with the dsRNA and NP signals (Figure 3C inset and Figure 3E). Quantitative analysis of the colocalization of dsRNA and p-PKR or p-PKR and NP in 100 randomly selected, infected cells indicated that the Pearson's correlation coefficient was 0.77 (Figure 3D) or 0.79 (Figure 3E), respectively. This suggests strong colocalization between dsRNA and p-PKR signals. These data support that dsRNA is recognized by and activates PKR in JUNV-infected cells.

### RLRs recognition of dsRNA in JUNV-infected cells

**Figure 4:**
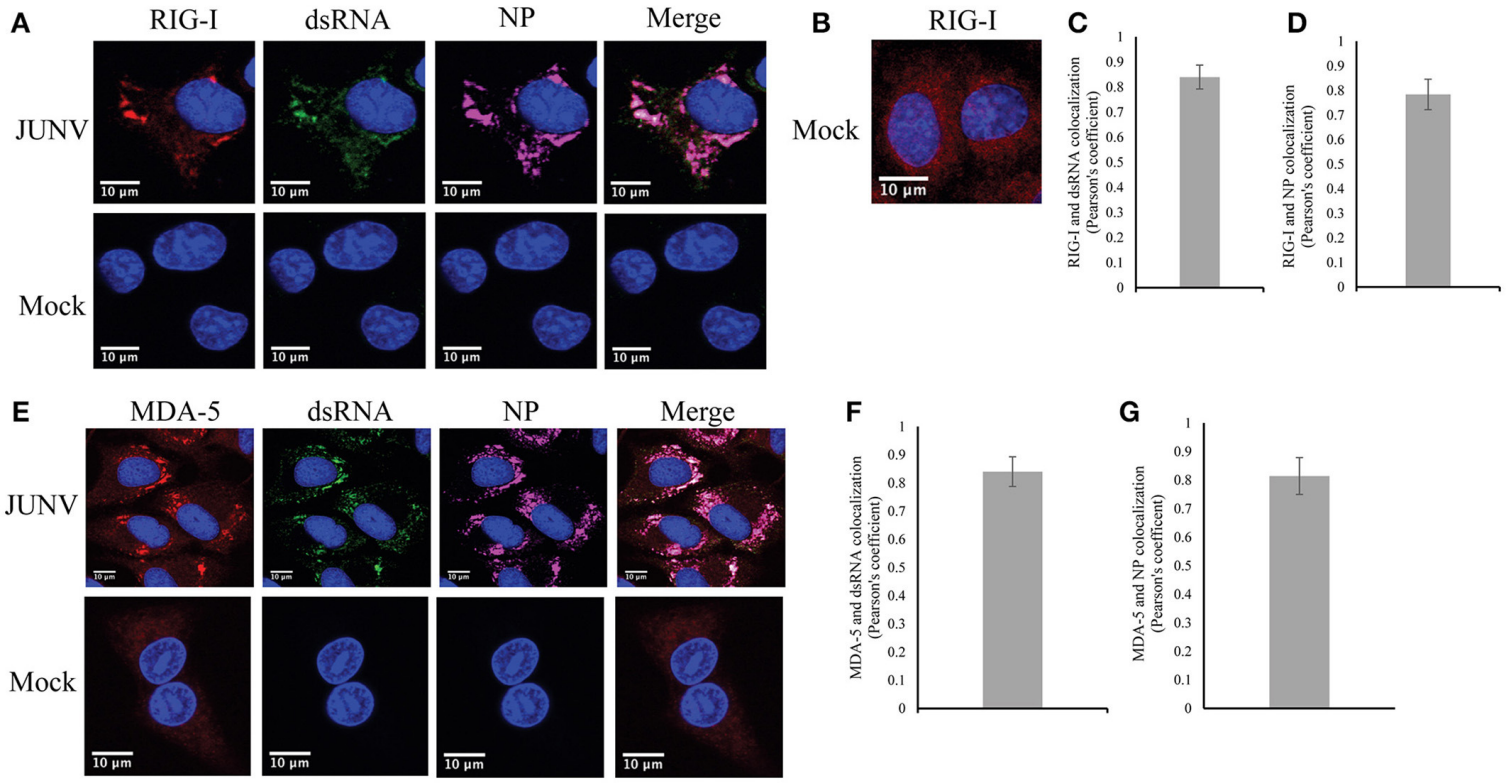
RLRs, RIG-I and MDA-5 recognize JUNV dsRNA. **(A,B,E)** A549 cells were infected with rCandid at a MOI of 1.0. 48 hpi infected and uninfected cell monolayers were fixed, permeabilized, and stained with the anti-dsRNA antibody (9D5, green), anti-JUNV NP (AG12, magenta), anti-RIG-I (red) **(A,B)** or anti-MDA-5 (red) **(E)** and DAPI (blue) as described in the Materials and Methods section. Cells were then observed under a Laser Confocal Scanning Olympus FV1000D Upright Microscope BX61 using 60x/1.42 numerical aperture oil immersion lens. All image analysis and processing was performed using the FIJI software. Unless indicated, laser emissions were the same for all samples and only the same linear adjustments for brightness and contrast were made when appropriate across all samples. Representative images of three separate experiment are shown. **(B)** Exposure of RIG-I in mock infected cells was increased to show the distribution pattern of RIG-I in uninfected cells. **(C,F)** Quantification of the colocalization between dsRNA and RIG-I **(C)** or MDA-5 **(F)** using the Pearson's correlation coefficient. **(D,G)** Quantification of the colocalization between NP and RIG-I **(D)** or MDA-5 **(G)**. Data shown represents the average and Std of 100 infected cells from three separate experiments.

Our previous data of the induction of a RIG-I-dependent type I IFN response in JUNV infected cells indicates the activation of RIG-I during NW arenavirus infection (Huang et al., 2012). RIG-I can recognize dsRNA structures (Jensen and Thomsen, 2012), which is present in the highly structured intergenic region and the pan-handle structure formed by arenavirus 5′ and 3′-ends of genomic RNA (Buchmeier et al., 2007). To determine if RIG-I can recognize dsRNA during NW arenavirus infection, the cellular localization of RIG-I, dsRNA and NP was studied in JUNV-infected A549 cells. The RIG-I levels were substantially increased in JUNV infected cells compared to mock (Figure 4A). To access the normal distribution pattern of RIG-I in mock-infected cells exposure was increased (Figure 4B) and RIG-I was found to be scattered throughout the cytoplasm. In JUNV infected cells, we found enrichment of RIG-I in areas containing punctuated structures of dsRNA and NP (Figure 4A). Quantitative localization analysis for dsRNA and RIG-I signals by measuring Pearson's correlation coefficient suggested a strong colocalization between dsRNA and RIG-I (0.84, *n* = 100) (Figure 4C), RIG-I and NP (Figure 4D) (0.79, *n* = 100) and dsRNA and NP (0.75, *n* = 100).

We next explored if dsRNA was recognized by another RLR, MDA-5, in JUNV-infected A549 cells by characterizing the cellular localization of MDA-5, dsRNA, and NP. Similar to the results of RIG-I, MDA-5 was upregulated in infected cells compared to mock infected cells (Figure 4E). MDA-5 exhibited a remarkable change in its distribution pattern from a diffuse localization in uninfected cells to the concentrated staining in infected cells (Figure 4E). The punctuated, often perinuclear, staining for MDA-5 colocalized with dsRNA and NP signals. The Pearson's correlation coefficient analysis for 100 cells suggested strong colocalization of dsRNA and MDA-5 (Figure 4F) (average of 0.84) and MDA-5 and NP (Figure 4G) (average of 0.81). Taken together, our data suggest that both RLRs, RIG-I and MDA-5, recognize dsRNA during NW arenavirus infection.

## Discussion

It is generally accepted that negative-sense RNA virus infections produce lower levels of dsRNA as compared with positive RNA virus infections. Many aspects of PRR recognition of virus derived dsRNA are still unknown for negative-sense RNA viruses. Indeed, we attempted to stain dsRNA with the widely used MAb J2 (Scion, Hungary) in JUNV-infected cells, but only observed background level signals that were non-differentiable from that in mock infected cells. The MAb 9D5 was originally developed for pan-enterovirus diagnosis. Recently, it has been established that the MAb 9D5 actually is a dsRNA-specific antibody, which does not react to ssRNA, ribosome RNA, tRNA and DNA, and has been used in detecting viral dsRNA including arenavirus LCMV dsRNA (Son et al., 2015; Child et al., 2018). MAb 9D5 seemed to be 20-times more sensitive than the MAb J2 in detecting dsRNA (Son et al., 2015). In our study MAb 9D5 readily detected dsRNA signal in NW arenavirus JUNV-infected cell (Figure 1A). Importantly, our RNase III treatment assay also confirmed the staining is specific to dsRNA (Figure 2), demonstrating that MAb 9D5 is a valuable tool to study host recognition of dsRNA in negative-sense RNA virus infection.

The viral origin of the dsRNA revealed in here remains to be identified in future studies. In our imaging study of JUNV-infected cells, the dsRNA signals largely colocalize with the viral NP puncta (Figures 1A,**B**), which is further supported by the quantitative Pearson's colocalization coefficient analysis (Figure 1C). The genomic and antigenomic RNA of arenaviruses are encapsidated by the NPs (Buchmeier et al., 2007). Previous studies also demonstrated the punctuated, often perinuclear staining pattern for NP in JUNV-infected cells (Baird et al., 2012; King et al., 2017a). It has been proposed that these structures contain virus replication-transcription complexes (RTC) as newly synthesized viral RNA was also detected in these cytosolic, NP-containing puncta in JUNV- and TCRV-infected cells (Baird et al., 2012). Most of viral mRNA does not seem to associate with viral RTC and are presumably transported outside from the RTCs (Baird et al., 2012). For LCMV, RNA FISH assay has also revealed strong colocalization of viral genomic RNAs with the aforementioned discrete NP structures as well as with the antigenomic RNA (King et al., 2017b). Thus, it is conceivable that some of the NP-associated dsRNA identified in our study in JUNV-infected cells are in close proximity to virus RTC and likely represent viral genomic/antigenomic RNAs or replication intermediates. We also noticed some of the dsRNA staining are in regions where the NP signal is scarce (Figure 1A), which might be derived from the viral mRNA populations that are transported outside the RTC as proposed (Baird et al., 2012).

To the best of our knowledge, our study is the first presenting the evidence of colocalization of dsRNA and NP during NW arenavirus infection (Figures 3, 4). The NPs of LASV, LCMV, TCRV, and PICV have been found to have a 3′-5′ exoribonuclease activity that preferentially digest dsRNA in biochemistry studies, an activity that has been proposed to enable arenavirus evade the innate immune response (Hastie et al., 2011; West et al., 2014; Huang C. et al., 2015). The DEDDh motif required for the exoribonuclease activity in NP is highly conserved among all arenaviruses (Qi et al., 2010; Hastie et al., 2011; Zhang et al., 2013; West et al., 2014; Huang C. et al., 2015). However, a study on the crystal structure of JUNV NP C-terminal domain showed that the NP could not incorporate Zn^2+^ and Mn^2+^ ions required for exonuclease activity for the DEDDh family and that the truncated JUNV NP could not digest dsRNA in a biochemistry assay (Zhang et al., 2013). This result is contradictory to an earlier study showing that the JUNV NP contains a zinc-finger motif and binds zinc ions (Tortorici et al., 2001). It should be noted that the truncated JUNV NP may function differently from the full-length NP. It is possible that JUNV NP somehow indeed lacks the exoribonuclease activity that may account for the dsRNA observed in proximity to NP in infected cells, or more likely, that dsRNA is produced and could be recognized by PRRs despite the exoribonuclease activity of JUNV NP. It is also interesting to investigate dsRNA formation for other pathogenic NW arenavirus, i.e., MACV, which also triggers potent IFN and PKR response during infection (Huang C. et al., 2015; Huang et al., 2017).

In the present study, we presented evidence of dsRNA production in NW arenavirus-infected cells and have characterized PRRs and dsRNA interaction by imaging analysis. Consistent with previous studies, our data support the notion that PRRs recognize viral dsRNA during NW arenavirus infection. Importantly, dsRNA colocalized with the phosphorylated, enzymatically active form of PKR in infected cells (Figure 3C), which clearly indicated PRR activation after sensing dsRNA. Our data opens new directions for studies on the interaction between arenavirus and host innate immune machinery. It remains to be determined whether the dsRNA-associated PRRs are functional in the presence of viral NP and Z proteins in infected cells. As the pathogenic NW arenavirus JUNV and MACV readily induce IFN response, apparently the NP and Z protein-mediated inhibition of IFN response is not absolute in the context of virus infection. Both the vaccine and pathogenic strains of JUNV stimulated IFN response in infected cells (Huang et al., 2012). Negrotto S et al. found that a pathogenic strain of JUNV stimulates higher levels of IFN production than the vaccine strain in human plasmacytoid dendritic cells (Negrotto et al., 2015). We also find similar results in human monocytes-derived dendritic cells (data not shown). The methodology we established in this study in BSL2 labs by using the vaccine JUNV could be applied in our future studies on highly pathogenic arenaviruses in BSL4 facilities, including the pathogenic strain of JUNV. As IFN response and PKR response are low or undetectable during OW LASV infection (Huang C. et al., 2015; Huang et al., 2017), future studies are warranted to study the formation and PRR recognition of dsRNA in LASV-infected cells.

## Author contributions

EM conceptualized the study, acquired the data, analyzed data, and assisted in manuscript preparation. CH and SP conceptualized the study, analyzed data, and assisted in manuscript preparation.

### Conflict of interest statement

The authors declare that the research was conducted in the absence of any commercial or financial relationships that could be construed as a potential conflict of interest.
